# Bioinformatics and Functional Analysis of an *Entamoeba histolytica* Mannosyltransferase Necessary for Parasite Complement Resistance and Hepatical Infection

**DOI:** 10.1371/journal.pntd.0000165

**Published:** 2008-02-13

**Authors:** Christian Weber, Samantha Blazquez, Sabrina Marion, Christophe Ausseur, Divya Vats, Mickael Krzeminski, Marie-Christine Rigothier, Rachid C. Maroun, Alok Bhattacharya, Nancy Guillén

**Affiliations:** 1 Institut Pasteur, Unité de Biologie Cellulaire du Parasitisme, Paris, France; 2 INSERM U786, Paris, France; 3 Institut Pasteur, Unité de Bio-Informatique Structurale, Paris, France; 4 Université de Paris-Sud, Faculté de Pharmacie, Laboratoire de Biologie et Contrôle des Organismes Parasites, Chatônay-Malabry, France; 5 Jawaharlal Nehru University, School of Life Sciences, New Delhi, India; 6 INSERM, Centre Paul Broca, Paris, France; Bose Institute, India

## Abstract

The glycosylphosphatidylinositol (GPI) moiety is one of the ways by which many cell surface proteins, such as Gal/GalNAc lectin and proteophosphoglycans (PPGs) attach to the surface of *Entamoeba histolytica,* the agent of human amoebiasis. It is believed that these GPI-anchored molecules are involved in parasite adhesion to cells, mucus and the extracellular matrix. We identified an *E. histolytica* homolog of PIG-M, which is a mannosyltransferase required for synthesis of GPI. The sequence and structural analysis led to the conclusion that EhPIG-M1 is composed of one signal peptide and 11 transmembrane domains with two large intra luminal loops, one of which contains the DXD motif, involved in the enzymatic catalysis and conserved in most glycosyltransferases. Expressing a fragment of the EhPIG-M1 encoding gene in antisense orientation generated parasite lines diminished in EhPIG-M1 levels; these lines displayed reduced GPI production, were highly sensitive to complement and were dramatically inhibited for amoebic abscess formation. The data suggest a role for GPI surface anchored molecules in the survival of *E. histolytica* during pathogenesis.

## Author Summary

The causative agent of the infectious disease, amoebiasis, is the parasite *Entamoeba histolytica,* which targets human intestine and liver. Once in the host, this parasite attaches to human cells and matrix components via factors at its surface such as the Gal/GalNAc lectin and proteophosphoglycans (PPGs). These factors are themselves anchored to the parasite surface by a glycosylphosphatidylinositol (GPI) moiety. To synthesise the GPI, a cascade of enzymes are necessary including the mannosyltransferase 1 (PIG-M1). A homolog of the PIG-M1 enzyme was shown to be present in *E. histolytica* (EhPIG-M1). To study the role of EhPIG-M1 in *E. histolytica*, parasites were constructed that had a reduced amount of mannosyltransferase. These parasites displayed a diminished production of GPI molecules and a lower amount of PPGs at the cell surface. Interestingly, the parasites were highly sensitive to the host blood complement and the formation of liver abscesses in hamsters was dramatically impaired. These results suggest that molecules anchored to the cell surface with the GPI moiety have a pivotal role in the survival of *E. histolytica* during pathogenesis.

## Introduction

Glycosylphosphatidylinositol (GPI) is a glycolipid required for anchoring many cell surface proteins and glycoconjugates to the surface of a wide range of human parasites including *Trypanosoma brucei*, the agent of sleeping sickness, *Leishmania* the causative agent of leishmaniasis, *Plasmodium falciparum* the agent of malaria and *Entamoeba histolytica* responsible for amoebiasis [1]. A common feature of the surface of these parasites is the presence of a large glycocalyx containing the GPI-anchored compounds that allow them to interact with their external environment. During invasion of human cells or tissues, the glycocalyx contributes to the adhesive mechanisms sustaining interaction of parasites with their target cells. GPI anchors are structurally complex glycophospholipids that are added to carbohydrate chains, as in the case of glycosylinositolphospholipids (GIPLs) and lipophosphoglycan (LPG) or post-translationally to the C-terminal end of many membrane proteins in the ER. Studies on the variant surface glycoproteins of *T. brucei* led to discovery of the role of GPI in anchoring proteins to the cell surface [2]. During parasitic infections, GPIs of various protozoan parasites, particularly those of *P. falciparum* and various Trypanosoma and Leishmania species, can activate host macrophages, triggering the production of proinflammatory cytokines and nitric oxide contributing to disease pathogenesis [1]. Recent studies have suggested that GPI and/or many GPI-anchored molecules could be secreted by the parasites during their invasive process. In the context of human infection by *P. falciparum*, it has been proposed that the secreted GPI of parasite origin functions as the dominant malarial toxin [3],[4]. GPIs of *T. cruzi* have the same function [5]. These data support the view that GPIs of the parasitic protozoa are dominant proinflammatory agents playing a role in the immunopathology of these parasitic infections. GPI-anchored molecules also play vital roles in amoebic pathogenesis. During dysentery, amoeba trophozoites bind to colonic mucins and to epithelial cells through the Gal/GalNAc lectin, an immunodominant protein complex containing a GPI-anchored subunit [6]. This lectin associates with another GPI-anchored protein, the intermediate IgL sub-unit. *E. histolytica* also expresses at its surface an abundant second class of GPI-linked molecules referred as GPI-anchored proteophosphoglycan (PPG) [7],[8],[9]. The GPI anchor of PPGs is unusual because it contains a highly acidic polypeptide backbone modified by 1-6 glucan side-chains and this core is also modified by heterogeneous galactose side-chains. Interestingly, the non-virulent *E. histolytica* strain Rahman synthesizes one class of PPGs containing short disaccharide side-chains [8] and no similar molecule was detected in the non-pathogenic species *Entamoeba dispar*. PPGs are important virulence factors during hepatic amoebiasis since monoclonal antibodies recognizing these compounds protect animals from abscess development [10]. The *E. histolytica* PPGs in addition diverge from the conserved sequence because they contain an anchor with the core structure Gal1Man2GlcN-myoinositol, where the terminal Gal residue replaces the 1-2 linked mannose residue of other eukaryotic GPIs. A large number of studies in yeast and mammalian cells allowed to conclude that steps in GPI biosynthesis are conserved in eukaryotes [11]. In general, biosynthesis of GPI begins in the Endoplasmic Reticulum (ER) with the transfer of N-Acetyl Glucosamine from UDP-N-Acetyl Glucosamine to ER membrane residing phosphatidylinositol (PI). This step is catalyzed by N-Acetylglucosamine transferase located in the ER membranes. This intermediate is then deacetylated to form GlcN-PI by another ER enzyme-GPI-deacetylase (PIG-L). It is thought that GlcN-PI is then flipped to the ER lumen by a set of flippases. Next, a set of mannosyltransferases acts on GlcN-PI to add on three mannose moieties successively to form (Man) 3-GlcN-PI [11]. This intermediate is recognized as a substrate by ethanolamine phosphotransferase to add on an ethanolamine phosphate group to the terminal mannose of the extending GPI glycan core, which is conserved in most eukaryotic cells. The mannose groups in the GPI core are all derived from dolichol-phosphate-mannose (Dol-P-Man). Thus, three Dol-P-Man dependent mannosyl transferases are required for independent addition of mannoses to the GPI-core. The first of these mannosyltransferases is PIG-M1 (Phosphatidylinositol glycan mannosyltransferase ) which transfers the first mannose to the growing GPI anchor from the luminal side [12]. The analysis of *E. histolytica* genome sequence allowed identification of genes involved in the GPI biosynthetic pathway [13]. Among a total of 22 genes in yeast and 23 in humans, 15 genes were identified in *E. histolytica*; these genes include all catalytic subunits of the enzymatic complexes sustaining GPI biosynthesis. Studies on the GPI biosynthetic pathway in *E. histolytica* are rather scarce. Nevertheless, it has been found recently that the antisense RNA-mediated inhibition of EhPIG-L, the GPI-deacetylase, has an important effect on cell growth, endocytosis and parasite adhesion to human cells [13].

In this report, we present the molecular identification in *E. histolytica* of PIG-M1. The analysis of the sequence of EhPIG-M1 reveals conserved residues that may play a role in the enzymatic catalysis and/or in the maintenance of the spatial structure of the protein. We demonstrated that anti-sense inactivation of mRNA encoding EhPIG-M1 leads to i) the accumulation of GlcN-PI intermediate; ii) a reduction of GPI contents on the amoeba surface; and iii) the inhibition of abscess formation in infected animals. The loss of parasite virulence phenotype may be due to an increase of complement sensitivity of the inactivated parasite strains.

## Materials and Methods

### Materials

UDP-[^14^C]N-acetyl glucosamine was obtained from Amersham, U.K. GDP-mannose, Dolichol mono phosphate (Dol-P), phosphatidylinositol, tunicamycin, tetracycline and mannosidase were from Sigma (USA). EN^3^HANCE spray for surface autoradiography was from Dupont, NEN, (France). HPTLC plates were from Merck. FLAER was obtained from Protox Biotech (Canada).

### Strains and culture conditions

Pathogenic *Entamoeba histolytica* (strain HM-1: IMSS) and derivatives were cultivated axenically in TYI-S-33 medium at 37°C [14]. Transfected trophozoites were maintained in presence of hygromycin at 5 µg ml^−1^ and the drug concentration was raised to 30 µg ml^−1^ for 48 h, then tetracycline was added at 1 µg ml^−1^ for 5 days before harvesting parasites.

Bacterial strain *Escherichia coli* TG1 was used for amplification of the plasmid constructions. Bacteria were grown in Luria-Bertani medium. Bacteria bearing plasmids were grown in presence of 50 µg ml^−1^ ampicillin.

### Gene identification and transcript extension

A cDNA fragment identified during EST program sequencing [15], was translated and the predicted peptide was compared by BLAST algorithm with the *E. histolytica* genome data base at TIGR. From growing trophozoites (10^7^) of the HM-1:IMSS strain, messenger RNA (mRNA) was prepared using Trizol reagent. To identify the mRNA encoded by *pigM1* gene a RACE experiment was performed according to manufacturer's protocol (Invitrogen, USA) using 5 g of total amoeba RNA. The reverse oligonucleotides used for 5′-RACE were designed on the XM_64 49 88 DNA sequence but are common to the two potential ORFs. For RT, ManT15 starting at base 650-5′AAA GCA GTT CCA AAT ACT GC; and for the nested PCR, ManT16 starting at base 581-5′AAA CAA AAG AAT AAT GGA AGT G. The forward oligonucleotide was in the anchored sequence added by RACE.

### Construction of an *E. histolytica* strain carrying an anti-sense fragment of pigM1 gene

The first 708 base pairs from the *E. histolytica pigM1* encoding gene (Accession number at NCBI: XM_644988) were amplified by PCR using the oligonucleotides 5′ GAG GAT CCA TGG GAA TAA AAG GTC AAG AAGG and 5′ CCA AAG AAA TTC GGT ACC ATA ACG ATA ATA AT that assure the insertion of a *BamH*I site at the 5′ end of the amplified fragment and a *Kpn*I site at its 3′ end. The amplified DNA fragment was cloned into the *Kpn*I-*Bam*HI sites of the Tet plasmid resulting in an inversion of this fragment compared to the oriented sense of the endogenous gene [16]. The resulting construct was verified by DNA sequencing and the recombinant plasmid was introduced in a virulent *E. histolytica* HM-1: IMSS strain recently passed through a hamster liver. As a control, the Tet vector containing the cat gene was also transfected. After five days of Tet treatment mRNA was purified with Trizol. Quality and integrity of purified RNAs was checked by spectrophotometry at 230, 260, 280 and 320 nm, electrophoresis on 0.8% agarose gel and assay on Bioanalyzer 2100 (Agilent, USA). Purified RNAs were retrotranscribed with Superscript II Retro-Transcriptase (Invitrogen, USA) according to manufacturer's protocol with specific primers detecting the sense or the antisense species of mRNA. In the same reaction mix was retrotranscribed the *L9* sense mRNAs (60S ribosomal protein L9-encoding gene), which was used as endogenous control.

### Computational sequence analysis of EhPIG-M1

We submitted ORF1 from *E. histolytica*, i.e., EhPIG-M1, to the GLOBE program (http://www.predictprotein.org/) for predicting the degree of globularity. GLOBE is based on figuring out the accessibility to the solvent. We then BLAST-searched the SwissProt and TrEMBL databanks for homologous sequences. From this output, we selected the sequences of six organisms (*H. sapiens* Q9H3S5; *R. norvegicus* Q9EQY6; *M. musculus* Q99J22/Q8C2R7; *C. elegans* Q17515; *D. melanogaster* Q9W2E4; *T. brucei* Q9BPQ5) for multiple alignments using the T-Coffee program [17] **.** Since these analyses indicated that the amino acid sequence of EhPIG-M1 corresponded to that of a membrane protein, we used the SignalP program [18] in order to predict the cleavage site of the sequence signal peptidase, i.e., the boundary between the signal peptide and the mature protein. To obtain the secondary structure content of the protein, we submitted the sequence to the PROFsec routine in the PHD suite of programs (http://www.predictprotein.org/).

We confronted the entire sequence to PROSITE, a database of protein families and domains (http://us.expasy.org/prosite/) in order to identify those sequence motifs that were conserved throughout the selected sequences and those that were proper to EhPIG-M1. For detection of the ER retention motif, we used the PSORT package [19].

### Prediction of transmembrane segments or Topography

We submitted the EhPIG-M1 sequence with and without the signal peptide to transmembrane prediction programs that have shown the highest accuracy in a recent comparative study [20]. These programs are TMHMM2 [21], HMMTop2 [22] and PHDpsihtm08 [23],[24]. The former two methods are based on hidden Markov models, whereas the latter is based on neural networks.

### Prediction of the orientation of the transmembrane segments or Topology

Once the transmembrane segments were predicted, we used the TopPred algorithm [25] for predicting the orientation of each transmembrane helix with respect to the membrane. The TopPred method makes use of the “positive-inside rule” which orients a transmembrane segment so that the difference in charge between the residues surrounding that segment and belonging to the intracytoplasmic and intraluminal media is positive. In addition, we calculated the hydrophobicity moment of each helix using the aWW scale [26], which takes into consideration the hydrophobic character of the amino acid residues, as well as the intra helical interactions between the side chains. Moreover, we also ran a prediction in which we imposed the following constraints: the N- and C-termini of the immature protein are intracytoplasmic, and the glycosylation site is intraluminal.

### Anti EhPIG-M1 antibody production

According to the amino acid sequence of EhPIG-M1, two peptides predicted to be located between two transmembrane domains were chosen for commercial antibody production (EUROGENTEC, Belgium). Two rabbits were injected with a mix of these peptides coupled to KLH. After four immunizations, sera from rabbits were tested by ELISA immunoreaction using the peptides and then specific IgG were purified by peptide affinity chromatography that uses the two peptides.

### Protein analysis of *E. histolytica* strains

Amoeba extracts from 10^6^ trophozoites were obtained after growing and washing the parasites with PBS and incubating the trophozite pellet in 100 µl of a protease inhibitor cocktail then boiled for 10 minutes. The extract was then mixed with Laemmli buffer and proteins were resolved in an 10% SDS-PAGE (equivalent to 10^4^ amoeba by lane). Resulting gels were Comassie bleu stained or used for immuno-detection. The preimmune serum (1∶500 dilution) and the peptide purified anti EhPIG-M1 (dilution 1∶2000) were used along with a secondary antibody (1∶25000) recognizing rabbit IgG (Jackson, USA) according to ECL revelation procedure (Amersham, UK). The primary anti-actin antibody (clone 4, Chemicon, USA) was used to normalize the protein loading and the ECL western blot image using the ImageQuant v.1.2 software (Molecular dynamics, GE Healthcare, USA). The ratio of EhPIG-M1 to actin allowed us to accurately assess variations in the quantity of EhPIG-M1 in the protein extracts. The experiments were performed twice.

### FLAER labelling of trophozoites to decorate GPI conjugate and LPG labelling

A million *E. histolytica* cells were labeled with 10^−8^ M concentration of FLAER (Protox Biotech, Canada) following Vats *et al*., 2005 [13]. For *in situ* epifluorescence labeling of PPG, 5×10^6^ parasites were incubated as described [10] using monoclonal antibody EH5 (1∶100 dilution) kindly provide by Dr Michael Duchene (University of Vienna, Austria). Fluorescent samples were examined on a Zeiss confocal laser scanning microscope with the pinhole fixed to 83 µm for all samples. Observations were performed in nineteen planes from the bottom to the top of each cell. The distance between scanning planes was 1 µm.

### Preparation of Crude Membranes and In vitro Biosynthesis of GPI pathway intermediates

Membranes were prepared from *E. histolytica* as described by Carver and Turco [27] and Vats *et al*, 2005 [13]. Crude membrane preparations (typically 1 mg protein) were used to analyse the GPI biosynthesis intermediates with ^14^C UDP N-acetylglucosamine (0.05Ci) in buffer [50 mM Hepes/NaOH (pH 7.5), 5 mM MgCl_2_, 5 mM MnCl_2_, 0.1 g/ml tunicamycin, 2M PI (phosphatidylinositol), GDP-Mannose (1.7M), Dolichol-P (20 g/ml), 1g/ml leupeptin, and 2mM PMSF], in a total volume of 200 l. Purification and analysis by thin layer chromatography (TLC) of intermediates was done according to Vats *et al*., 2005 [13]

### Hepatic inoculation procedure and preparation of infected liver sections

Male Syrian golden hamsters (*Mesocricetus auratus*), aged 6 weeks, with an average weight of 100 g, were inoculated intraportally, intrahepatically or intraperitonial with 5×10^5^ HM-1:IMSS trophozoites, anti-sense EhPIG-M1 and control strains [28]. Drinking water provided to these animals was supplemented with 50μg/ml of tetracycline 48 hours before inoculation and during the entire infestation procedure. Hamsters were sacrificed 7 days after inoculation. The livers were removed, inspected for the presence of amebic abscesses and photographed.

### Measurement of complement resistance of virulent HM-1:IMSS and EhPIG-M1 antisense strains

Lysis of 4×10^6^ parasites by hamster complement was determined on wild type pathogenic HM-1:IMSS strain, PIG-M1-AS or control transfected strains cultured in presence of tetracycline. Hamster serum was used as the complement source and heat-inactive serum (56°C, 30 min.) was prepared as a control. Serum concentration was fixed at 30% of the final volume; a concentration that is not toxic for virulent strains. Three different times of contact were experimented: 15, 30, 60 min. Twenty l of trophozoite suspension were mixed in 96 wells plate with 30 l of serum and 50 l of PBS containing 2,5 mM MgCl_2_, 10 mM EGTA in a final volume of 100 µl; presence of Mg/EGTA activate the alternative pathway. Control, heat-inactive serum, was assayed in the same conditions. The assay mixtures were incubated at 37°C. Samples were removed at each defined time interval and resting intact trophozoite were counted using Malassez chamber and phase contrast microscopy.

The percentage of lysis resistance was calculated as = % of complement resistance = N_s_/N_dc_×100. Samples were analyzed in duplicate and the experiments were repeated three times. (N_s_; number of viable trophozoites after contact with fresh serum, N_dc_: number of viable trophozoites after contact with decomplemented serum). Statistical parameters were determined using a two-way analysis of variance (ANOVA). Inter-strain differences were tested using Fisher's multiple mean comparison method.

## Results

### Identification and molecular analysis of EhPIG-M1 from *E. histolytica*

Large scale sequencing of transcripts from *E. histolytica* [15] allowed us to identify a cDNA fragment that when translated predicted a polypeptide sharing a significant level of sequence similarity with α1,4 mannosyltransferases (PIG-M) from humans. BLAST-based sequence similarity search with the *E. histolytica* genome revealed two homologues of PIG-M. The locus 1 (XM_644988) displays 38% identity to human PIG-M across the entire length of 412 amino acids. The second locus (XM_647339) encodes a protein of 384 amino acids, out of which a block of 379 amino acids show 97% identity when compared with locus 1. Both putative proteins have the DXD motif. This motif is thought to be involved in binding a manganese ion necessary to interact with the nucleotide sugar substrate, a characteristic of glycosyltransferases [29],[30]. It is generally present in mannosyltransferases. However, the two putative genes differ in their overall organization, such as sequence divergence at 5′ UTR regions and presence of an intron in ORF2. There is a stop codon at amino acid position 226 which falls within the predicted short 9bp intron of ORF2. The predicted intron, in fact, removes the stop codon giving a longer ORF. In order to determine whether or not these two genes were expressed, we used primers allowing for amplification of the 5′ end of both the putative mRNAs by a RACE experiment. Purified mRNA from growing parasites was converted to cDNA following a primer extension approach. The amplified DNA fragment was purified and the nucleotide sequence was determined. The results indicated that only the DNA fragment encoding the ORF1 was transcribed during parasite culture; the product of ORF 1 was then named EhPIG-M1. The data suggests that EhPIG-M1 gene may be expressed by exponentially growing *E. histolytica.* It is difficult to say at present if locus 2 is a pseudogene or not as it may get expressed under conditions not tested by us.

### In silico analysis of EhPIG-M

**Figure 1:**
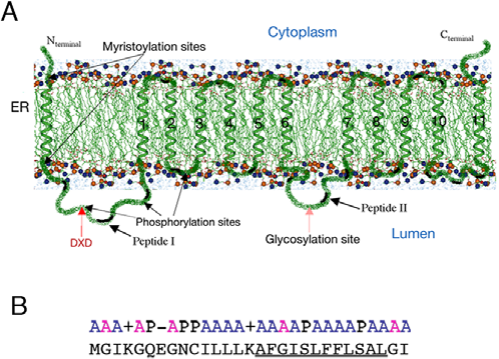
Predicted 2D structural arrangement of EhPIG-M1 from *E. histolytica.* A. The protein is presented in a segment of the ER membrane. Beside the helical signal peptide, 11 transmembrane (TM) domains are predicted. These domains and the amino acids that they span are TM1: 99-115, TM2: 123-140, TM3: 145- 161, TM4: 168-184, TM5: 189-205, TM6: 212-228, TM7: 272-288, TM8: 295-312, TM9: 317-333, TM10: 340-359, and TM11: 375-399. Several potential post-translational modification sites are shown. On the luminal side the loop carrying the DID motif and peptides I and II are indicated by an arrow. B. The signal peptide of EhPIG-M1. The signal peptide of 29 amino acids is organized in two domains: a polar domain carrying two positive and one negative charges, followed by a hydrophobic domain. The polar C-terminal domain typical of signal peptides is missing. A: non-polar, P: polar. The central hydrophobic domain is underlined.

The two-dimensional structural organization of EhPIG-M1 was deciphered by using a number of bioinformatics tools as described in experimental procedures. The overall predicted organization of the protein with respect to the lipid bilayer membrane is shown in Figure 1A. EhPIG-M1 is a polytopic membrane protein likely to be associated with the ER/Golgi system. A signal peptide is present at the N-terminus. The model produced by HMMTop2 in absence of the signal peptide sequence gave equivalent results whether the topographic constraints (N- and C-terminal extremities intra-cytoplasmic and glycosylation site intraluminal) were introduced or not. EhPIG-M1 is composed of 12 transmembrane spans -the signal peptide and 11 transmembrane helices with two large intraluminal loops, O1 and O4 (Figure 1A and Figure S1). The N-terminus of the immature protein is intra-cytoplasmic, in agreement with the known mechanisms of protein translocation in the ER [31],[32]. The signal peptide transmembrane fragment is predicted to be in a helical conformation. The first loop (O1, residues M31-I98) in the immature protein contains the DXD motif. The release of the N-terminal anchor point upon release of the signal peptide should facilitate the folding of this fragment. Surprisingly, the EhPIG-M1 sequence presents no Carbohydrate Recognition Domain (CRD) that could interact with mannose. In addition, we find no motif consisting of two glutamates separated by seven residues, motif whose importance for the enzymatic activity of glycosyltransferases has been recognized. Two conserved amino acids surround the DXD motif–a threonine and a tyrosine. The extended ^45^TDIDY^53^ motif corresponds to a potential phosphorylation site, possibly involved in regulating the catalytic act. According to the aWW scale, the transmembrane helices in EhPIG-M1 are in general hydrophobic, rather than amphipathic. In our 2D model, the following pairs of helices are linked by small loops, mostly hydrophilic and, being geometrically constrained must closely pack: 1-2, 2-3, 3-4, 4-5, 5-6 and 7-8, 8-9, 9-10, 10-11. Thus because the O4 loop (E229-K267) between transmembrane helices T6 and T7 is rather large, the transmembrane helices in EhPIG-M1 may consist of two structural domains, one composed of helices 1-6 and the other of helices 7-11 (Figure 1A). The topology of the proposed model in the usual notation is as follows: (cytosol) Nter-(single TM)-GD-(multiple TM)-GD-(multiple TM)-Cter, TM representing the transmembrane passages and GD the globular domains. The overall hydrophobicity of most of the passages leads us to believe that thay will have a tendency to conglomerate

### Identification of EhPIG-M1and functional role in *E. histolytica*

**Figure 2:**
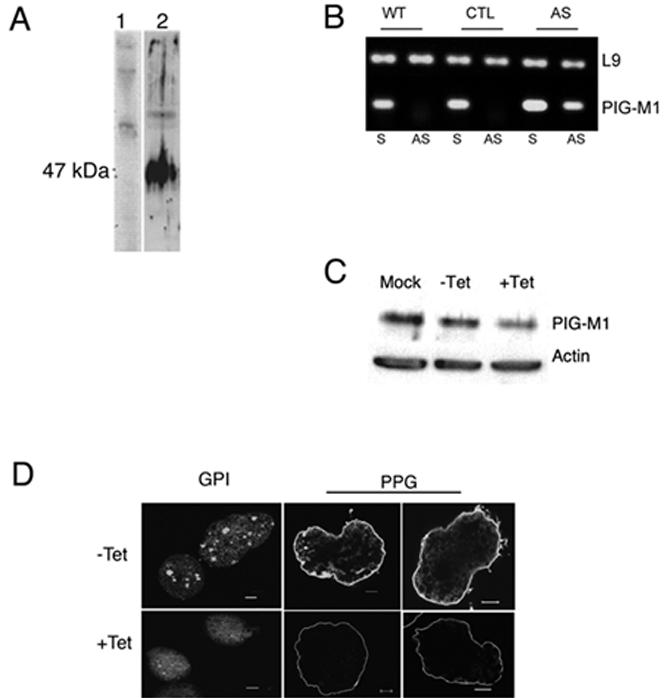
Expression of EhPIG-M1 in *E. histolytica* WT and anti-sense strains. A. Protein analysis by immunoblot of extracts of wild type parasites. Electrophoretic resolution in 8% PAGE and immunoblots of proteins from a crude extract (equivalent to 10^4^ cells by lane). Proteins were probed by immunoblot with pre-immune serum (lane 1) and with a anti-EhPIG-M1 antibody (lane2). B. Cloning and expression of anti-sense mRNA expressing parasites. The fragment cloned in anti-sense carries the first 708 bp of XM_64 49 88 loci. Agarose gel electrophoresis of PCR amplified products showed an unique DNA fragment (56 bp) amplified with antisense detecting primers when RNA was purified from PIG-M-AS strain. Antisense RNA was indeed absent from the wild type strain (WT) and from the control strain (CTL), which carries the vector. All strains allow for amplification of the sense mRNA. L9 correspond to ribosomal protein L9 encoding mRNA used as a control (81 bp). No amplification was detected for the two amplicons when RT was absent in the assay. S = sense detecting primers, AS = anti-sense detecting primers. C. Western blot analysis of protein lysates separated in SDS-PAGE. Extract from PIG-M-AS parasites (+/− tetracycline) were used (equivalent to 10^4^ cells by lane). EhPIG-M1 is at 47 kDa, and actin used as a blot reference at 43 kDa. The ECL film image was treated with ImageQuant software. D. Labeling of GPI-linked molecules by FLAER and PPG by immunofluorescence. PIG-M-AS cells grow with or without Tet were incubated with FLAER and treated for confocal microscopy. Upper left panel: FLAER labeled uninduced cells. Lower left panel: FLAER labeled induced cells expressing antisense mRNA, notice the loss of vesicle labeling. To localize PPG , PIG-M-AS trophozoites growing in the presence (or not) of tet for 5 days were fixed and incubated with specific mAb-5 recognizing PPG and treated for confocal microscopy. The micrograph (taken at the middle of the cell) shows a reduction of PPG labeling when the AS-cells were treated with tet. Notice the lost of spike-like structures around the cell. Bar = 5 m.

The 2D model of EhPIG-M1 inferred (Figure 1A), predicts that the two internal peptides ^64^VNGESPYRRATYRYTPL^80^ (peptide I) and ^238^TYLYHGTRTDHRHNL^252^ (peptide II) do not belong to any membrane domain. Taking into account this sequence information, peptides I and II were synthesized and used to prepare an anti-EhPIG-M1 antibody (precisely amino acids 62 to 74 and 232 to 244). For that purpose; rabbits were immunised with a mixture of these peptides in order to raise specific antibodies. The purified antibody was used in immunoblots to investigate the presence of EhPIG-M1 in *E.histolytica* protein extract. A unique polypeptide of apparent molecular mass of 47 kDa was identified (Figure 2A). This matched the expected mass of the EhPIG-M1 protein. To investigate the potential role of EhPIG-M1 in *E. histolytica* pathogenesis we constructed a parasite strain transcribing an antisense RNA of the first 708 nucleotides of the EhPIG-M1 encoding gene using a tetracycline (tet) inducible gene expression system [16]. We first determined whether the plasmid construct induced transcription of an antisense EhPIG-M1 RNA in parasites treated by tet. RT-PCR analysis showed the presence of a specific band indicating the presence of AS-RNA (Figure 2B). The level of EhPIG-M1 protein in transfected cells was analysed by a western blot assay and the results showed that the abundance of EhPIG-M1 in the antisense expressing parasites was reduced to 60% (Figure 2C) of that from control parasites. These data suggest that induction of anti-sense RNA leads to reduction of EhPIG-M1 cell content.

To investigate a consequent potential reduction in GPI content, FLAER labeling was done on living PIG-M-AS trophozoites, incubated with or without tet and examined by confocal microscopy. There was roughly 4-5 fold decrease of GPI- labeling by FLAER in tetracycline induced PIG-M-AS cells against uninduced cells (Figure 2D). Differential labeling was particularly seen at the level of the number of fluorescent vesicles within a cell. Induction of antisense RNA by tet significantly reduced the number of fluorescent vesicles suggesting a reduction of the amount of GPI within the cell. Since one of the most abundant GPI-anchored molecule at the *E. histolytica* surface is proteophosphoglycan (PPG), we investigated whether changes in overall PPG content appeared subsequently to reduction of EhPIG-M1 level. Using confocal microscopy and monoclonal anti-PPG antibody EH5 (mAb5), we observed a low but significant reduction of PPG on the amoeba surface (Figure 2D). However, there was no detectable change in the level of Gal/GalNAc lectin light subunit (anchored by GPI on the amoebic surface) in tet induced EhPIG-M1 antisense cells (data non shown). There was also no significant difference in the level of phagocytosis of red blood cells or surface receptor capping activity on tet induction in these cells (data non shown) suggesting that these two phenomena were not modified by the reduction of EhPIG-M1 levels.

### Characterization of GPI-intermediates in Eh PIG-M1 antisense expressing cells

**Figure 3:**
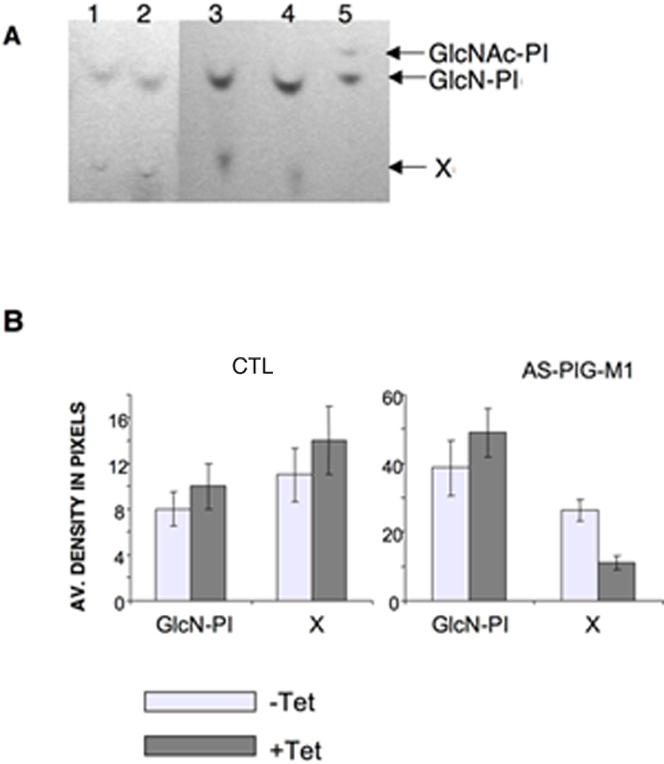
GPI-intermediate biosynthesis with extracts from EhPIG-M1 antisense blocked cells. Crude membrane preparation from tetracycline induced and uninduced trophozoites were labeled with [14]-C UDP-N-acetylglucosamine in a cell free system in the presence of GDP-mannose. GPI biosynthesis intermediates were isolated and analyzed by HPTLC and visualized by fluorography. Panel A, Lane 1-2: GPIs from untreated and tetracycline treated vector control respectively, lane 3-4, GPIs from untreated and tetracycline treated EhPIG-M1 antisense cells line respectively; lane 5 shows the first two intermediates of the GPI-pathway, namely GlcNAc-PI and GlcN-PI, as control. Panel B shows the densitometric analysis of the radiolabeled products as obtained on HPTLC chromatography, I- GlcN-PI and product X for untreated (clear boxes) and tetracycline treated (black boxes) cells carrying the control vector (CTL), or the EhPIG-M1 antisense construct. An increased level of GlcN-PI (the substrate of PIG-M) is observed in extract carrying reduced levels of EhPIG-M1.

If EhPIG-M1 encodes a functional α1,4 mannosyltransferase, then inhibition of its expression by antisense RNA should lead to an accumulation of the GlcN-PI intermediate, a substrate for PIG-M. A decrease in the synthesis of Man-GlcN-PI (product of PIG-M activity) is also expected. The first two intermediates of GPI-biosynthesis have been previously characterized [13] as GlcNAc-PI and GlcN-PI, stressing on the functional existence of the GPI biosynthesis pathway in *E. histolytica*. Therefore, to validate the functional aspect of EhPIG-M1 we set up an *in vitro* reaction with crude membrane preparation and labelling with [^14^C] UDP-N-acetylglucosamine the intermediate products of GPI biosynthesis. The radiolabeled GPIs synthesized by the parasite extract were isolated and quantified in TLC as previously described [13]. Previously isolated and characterized GlcNAc-PI and GlcN-PI were taken as control and migration references. Addition of GDP-Mannose and Dolichol-P resulted in the formation of a new species X, which migrated slower than GlcNAc-PI and GlcN-PI (Figure 3A). Since no other radiolabeled spots were obtained besides GlcN-PI and X, we concluded that GlcN-PI was processed to X. Densitometric analysis showed that there was a decrease of roughly 50% in the production of X compound when membranes from tet-induced antisense parasites were used and compared to the non induced set (Figure 3B). The data suggests that EhPIG-M1 may be using GlcN-PI as a substrate producing X, which is probably the mannosylated form of GlcN-PI. In correlation, GlcNAc-PI accumulates in the extract from EhPIG-M1-AS strain.

### Reduction of GPI inhibits virulence and complement resistance in *E. histolytica*

**Figure 4:**
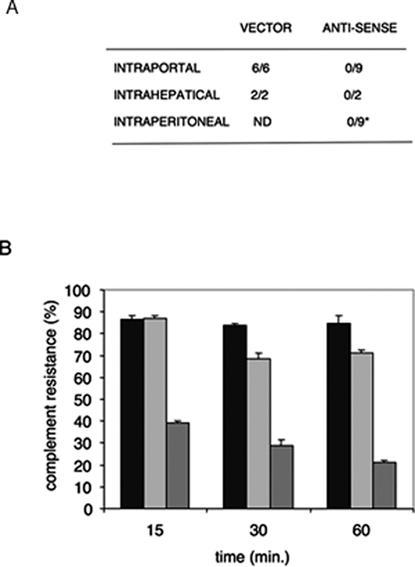
Pathophysiological analysis of hamster liver after infection and complement resistance with EhPIG-M1-AS parasites. A. Summary of different sets of infection by several routes. Notice the absence of abscesses when the infection was performed with the EhPIG-M1-AS strain induced for antisense RNA production compared with the controls. Animals (EhPIG-M1-AS strain infected) were fed with tetracycline three days prior to the inoculation and during the seven days of the experiment. B. Resistance of parasites to blood complement. The parasites from the wild type strain (black columns, (WT), and tetracycline treated control (lightly grey columns, CTL) and tetracycline treated anti-sense (grey columns, AS) strains were incubated in the presence of hamster serum diluted to 30%. Entire cells were counted under the microscope. Serum heated at 56¡C (heat inactivated) was used as a control showing 100% survival of all kind of parasites. Notice that the strain carrying the anti-sense RNA was significantly lysed compared with the control (plasmid vector) and with the wild type strain.

The virulence phenotype of EhPIG-M1-AS cell line was examined by using the liver abscess model in hamsters with and without tet induction. Animals were infected with PIG-M-AS cells that were cultivated for five days in the presence of tetracycline. A control was performed by injection of animals with amoebas carrying the vector plasmid, with the cat gene treated in the same manner. Infected hamsters drank water containing tetracycline for the period of infection and then they were sacrificed for pathophysiology analysis. A dramatic reduction of pathogenicity was observed (Figure 4A). In contrast to the controls, abscesses were not seen in animals infected with EhPIG-M1-AS parasites in spite of infection carried out using different routes, that is, intrahepatical, intraperitonial or intraportal. A histological observation of hepatical lesion of tissue in the area of injection demonstrated, by comparison with the controls, that EhPIG-M1-AS trophozoites did not survive in the living tissue. We then used live imaging by two-photon microscopy to follow PIG-M1-AS strain during infection in real time. We observed that tet-induced PIG-M1-AS cells were completely lysed as soon as these trophozoites were in contact with the animal tissue. The sensitivity of PIG-M1-AS strain to living tissue factors precluded any further video-microscopy analysis and prompted us to measure the behaviour of these parasites in the presence of hamster serum, rich in complement.

To test the parasite survival in the presence of serum, trophozoites from the wild type strain, the control and EhPIG-M1-AS cells grown in the presence and the absence of tet were incubated with 30% of hamster serum for different periods of time. A large destruction of EhPIG-M1-AS strain expressing the antisense construct was observed within 15 minutes of incubation (Figure 4B) whereas the WT and the non-induced PIG-M1-AS cells were resistant (p<0.001). All the parasite cells were insensitive to heat inactivated serum with no complement activity. Overall the results suggest that reduction of EhPIG-M1 by anti-sense technology is able to reduce cell surface GPI molecules and accounts for inhibition of important pathogenicity features such as resistance to complement and ability to cause liver abscess.

## Discussion

In this work we have identified an *E. histolytica* protein homolog of mammalian PIG-M, an endoplasmic reticulum–localized α1,4 mannosyltransferase required for synthesis of the glycosylphosphatidylinositol (GPI) anchor. PIG-M is the enzyme that incorporates the first mannose into the GPI core [11]. The EhPIG-M1encoding gene (XM_644988) is transcribed in growing parasites as assessed by RACE-PCR experiments and by cDNA sequencing. Bioinformatics analysis of EhPIG-M1 secondary structure reveals that it is a transmembrane protein probably residing in the ER. The C-terminus containing the retention motif is intra-cytoplasmic indicating that it can be recognized by COP I, a specific cytoplasmic protein that retrieves proteins from the Golgi after interaction with the ER retention motif [33],[34]. However, the potential ER retention motif -^403^LRKQKQLKLN^412^- is atypical. Since the organization of the ER and Golgi of this early branching eukaryote *E. histolytica* is different from other eukaryotes it is possible that the ER retention signal may be different [33],[34]. The cellular location and trafficking of EhPIG-M1 are matters under investigation.

There are a few successful examples of functional characterization of *E. histolytica* genes using antisense RNA [13]. This approach is particularly useful in absence of a method available to carry out targeted gene deletion. In this study, the antisense RNA approach has been used to understand the role of EhPIG-M1 in amoebic virulence. This strategy generated parasites with 40% reduction in EhPIG-M1 content that leads first to the accumulation of GlcN-PI, the substrate of EhPIG-M1, indicating that there is a loss of activity of this enzyme. These parasites also displayed a radical phenotype shown by dramatic changes in their survival when incubated in the presence of complement and are non virulent in the hamster model of hepatical amoebiasis. The reduction of GPI and/or GPI-anchored molecules at the amoeba surface observed by staining should account for these phenotypical changes leading to loss of virulence due to their enhanced susceptibility to complement action (Figure 4).

The human complement system, as part of the humoral innate immune system, is essential for recognition of microbes, opsonization followed by intracellular killing by phagocytes, or direct lysis. Previous works have shown that the extracellular cysteine proteinases of *E. histolytica* activate the complement pathway by specifically cleaving C3 leading to a modified C3b-like protein thus preventing terminal membrane attack complex (MAC) formation [35]. This interesting mechanism of complement activation (which should appear as a disadvantage for the survival of the parasite), leads to passive lysis of non-pathogenic strains; whereas pathogenic strains are resistant to complement attack [36]. Moreover, the Gal/GalNAc lectin, present on the amoeba surface, inhibits the assembly of lytic MAC [37]. Complement binds to *E. histolytica* surface and it is believed that receptors for complement molecules exist in this parasite. However, the mechanism by which amoeba resist complement action is not well known and whether GPI-anchored molecules participate in this feature remains to be molecularly established. However, examples for the involvement of GPI-anchored molecules in complement resistance have come from *Leishmania*. This parasite resists by prevention of binding at their surface of the attack complex. LPG, a GPI-anchored glycoconjugate, is a major C3 binder [38]. MAC is formed on the top of the LPG coat and does not reach the membrane bilayer leading to its spontaneous elimination. Interestingly, a *Leishmania major* lpg1- mutant, which lacks LPG, shows attenuated virulence and is highly susceptible to human complement, indicating that LPG can act as a biological barrier thus protecting parasites from complement attack [39]. The experiments reported here suggest one important role of GPI and of GPI-containing molecules for *E. histolytica* pathogenesis. Increasing our knowledge of GPI biosynthetic pathway in this pathogen will open opportunities for the discovery of alternative treatments against amoebiasis.

## Supporting Information

Alternative Language Abstract S1. **Translation of the abstract into French by Nancy Guillén.**
(0.03 MB DOC)

Alternative Language Abstract S2. **Translation of the abstract into Spanish by Samantha Blazquez.**
(0.03 MB DOC)

Figure S1. **Amino acid sequence homology between PIG-M from *E. histolytica* and PIG-M from other eukaryotes.**
The amino acid sequences were retrieved from the National Center for Biotechnology Information (NCBI) libraries and compared by BLAST. *E. histolytica* (XM_644988); *H. sapiens* (Q9H3S5); *R. norvegiens* (Q9EQY6); *M. musculus* (Q99J22); *C. elegans* (Q17515); *D. melanogaster* (Q9W2E4); *T. brucei* (Q9BPQ5). Alignment was done using the T-Coffee program [16]. The following potential sites are common to all the sequences: two sites of phosphorylation by phosphokinase C (^74^TYR^76^ and ^141^STR^143^); one site of phosphorylation by casein-kinase 2 (^49^TDID^52^); two sites of N-myristoylation (^5^GQEGNC^10^ and ^28^GIRMGL^33^) that surround the hydrophobic region of the ER sorting signal sequence; one site of O-glycosylation (^251^NLSV^254^). In addition, the potential sites specific to the *E. histolytica* sequence are: two phosphokinase C phosphorylation sites (^269^TIR^271^ and ^402^SLR^404^); one AMPc- and GMPc-dependent protein kinase phosphorylation site (^71^RRAT^74^); three casein kinase 3 phosphorylation sites (^101^SFID^104^, ^244^TRTD^247^ and ^398^SLSD^401^); one tyrosine-kinase phosphorylation site (^47^RYTDIDY^53^); and one N-myristoylation site (^144^GNAEAV^149^). I: intracytoplamic loop; O: intraluminal loop; T: transmembrane helix.
(0.05 MB DOC)
