# Supplementary material for: Bioinformatics and Functional Analysis of an Entamoeba histolytica Mannosyltransferase Necessary for Parasite Complement Resistance and Hepatical Infection
Source: PLoS Negl Trop Dis. 2008 Feb 13;2(2):e165. doi: 10.1371/journal.pntd.0000165 (PMC2239303; doi:10.1371/journal.pntd.0000165)
Supplement: Alternative Language Abstract S1. — Translation of the abstract into French by Nancy Guillén. (0.03 MB DOC) [file pntd.0000165.s001.doc]

Résumé en français

L’agent responsable de l’amibiase est le parasite *Entamoeba histolytica* qui colonise l’homme au niveau intestinal et hépatique. A l’intérieur de tissus de l’hôte, le parasite s’attache aux cellules humaines et à la matrice extracellulaire grâce à des facteurs de surface comme la lectine Gal/GalNAc et le proteophosphoglycane (PPGs). Ces composants sont ancrés à la surface du parasite par leur groupement glycosylphosphatidyl-inositol (GPI). La synthèse du GPI nécessite une série d’enzymes dont la mannosyltransferase 1 (PIG-M1). Dans ce travail, un homologue de PIG-M1 a été retrouvé chez *E. histolytica* (Eh PIG-M1). Afin d’étudier le rôle de EhPIG-M1 dans le processus pathogène, une souche de parasite exprimant un faible niveau de mannosyltransferase 1 a été construite. Ces parasites présentent une diminution du nombre de molécules GPI et une plus faible quantité de PPGs. De façon intéressant ces parasites sont très sensibles au système du complément et leur capacité à former des abcès hépatiques chez le hamster est très fortement réduite. Ces résultats suggèrent que les molécules ancrées à la surface du parasite au moyen du GPI ont un rôle important dans la survie de *E. histolytica* pendant sa phase invasive.
