# Supplementary material for: Bioinformatics and Functional Analysis of an Entamoeba histolytica Mannosyltransferase Necessary for Parasite Complement Resistance and Hepatical Infection
Source: PLoS Negl Trop Dis. 2008 Feb 13;2(2):e165. doi: 10.1371/journal.pntd.0000165 (PMC2239303; doi:10.1371/journal.pntd.0000165)
Supplement: Alternative Language Abstract S2. — Translation of the abstract into Spanish by Samantha Blazquez. (0.03 MB DOC) [file pntd.0000165.s002.doc]

Resumen en Español

El parásito *Entamoeba histolytica* es el agente microbiano responsable de la amibiasis humana que se caracteriza por la colonización del intestino y ocasionalmente del hígado. Una vez instaladas en el huésped, las amibas reconocen y se asocian a las células blanco y a la matriz extracelular utilizando factores propios de superficie tales como la lectina Gal/GalNac y los proteofosfoglicanos (PPGs). Estos compuestos son expuestos en la superficie del parasito mediante un ancla de glicosilfosfatidilinositol (GPI). La biosíntesis de GPI requiere varias enzimas entre las cuales destaca la manosiltranferasa 1 (PIG-M1). En este trabajo hemos identificado una enzima homologa de PIG-M1 en *E. histolytica* (EhPIG-M1). Con el objetivo de estudiar el papel de EhPIG-M1 durante la patogénesis, hemos construido une cepa de amibas que expresan un nivel reducido de manosiltransferasa. Esta cepa de amibas deficientes en EhPIG-M1 también expresan menos GPI y por consecuente menos PPGs. Ensayos biológicos muestran que las amibas deficientes en EhPIG-M1 son susceptibles a la muerte mediante la acción del complemento sanguíneo y además son incapaces de formar abscesos hepáticos en el modelo de amibiasis en el hámster. Los resultados muestran que las moléculas expuestas en la superficie del parasito mediante GPI, juegan un papel importante en la sobrevivencia de *E. histolytica* durante la fase invasiva.
