# Supplementary material for: Bioinformatics and Functional Analysis of an Entamoeba histolytica Mannosyltransferase Necessary for Parasite Complement Resistance and Hepatical Infection
Source: PLoS Negl Trop Dis. 2008 Feb 13;2(2):e165. doi: 10.1371/journal.pntd.0000165 (PMC2239303; doi:10.1371/journal.pntd.0000165)
Supplement: Figure S1. — Amino acid sequence homology between PIG-M from E. histolytica and PIG-M from other eukaryotes. The amino acid sequences were retrieved from the National Center for Biotechnology Information (NCBI) libraries and compared by BLAST. E. histolytica (XM_644988); H. sapiens (Q9H3S5); R. norvegiens (Q9EQY6); M. musculus (Q99J22); C. elegans (Q17515); D. melanogaster (Q9W2E4); T. brucei (Q9BPQ5). Alignment was done using the T-Coffee program [16]. The following potential sites are common to all the sequences: two sites of phosphorylation by phosphokinase C (74TYR76 and 141STR143); one site of phosphorylation by casein-kinase 2 (49TDID52); two sites of N-myristoylation (5GQEGNC10 and 28GIRMGL33) that surround the hydrophobic region of the ER sorting signal sequence; one site of O-glycosylation (251NLSV254). In addition, the potential sites specific to the E. histolytica sequence are: two phosphokinase C phosphorylation sites (269TIR271 and 402SLR404); one AMPc- and GMPc-dependent protein kinase phosphorylation site (71RRAT74); three casein kinase 3 phosphorylation sites (101SFID104, 244TRTD247 and 398SLSD401); one tyrosine-kinase phosphorylation site (47RYTDIDY53); and one N-myristoylation site (144GNAEAV149). I: intracytoplamic loop; O: intraluminal loop; T: transmembrane helix. (0.05 MB DOC) [file pntd.0000165.s003.doc]

**Figure S1**

.........................1........10........20.........30........40........50

| | | | | |

H.SAPIENS...........-----MGSTKHWGEWLLN-LKVAPAGVFGVAFL-ARVALVFYGVFQDRTLHVRYTDIDYQ

R.NORVEGICUS........-----MSYTKHWGEWFLN-LRVPPAGVFGVAFL-ARVALVFYGVFQDRTLLVRYTDIDYH

M.MUSCULUS..........-----MSYPMHWGEWILN-FRVPPAGVFGVAFL-ARVALVFYGVFQDRTLLVRYTDIDYH

C.ELEGANS...........--------MQCVRSFVKN-ETFNRNKILLVAFV-ARIILVFYAHIHDYLFKVNFTDIDYH

D.MELANOGASTER......MTQTKAATTGWQRLGR-RILYTSFRTHLLISAL-LRIALICYGQLHDSQSAVPYTDIDYK

T.BRUCEI............-----------MELQSLI-DTVSLQKLLLLGAL-LRLILIAYAFFHDQWFRVKYTDIDYM

E.HISTOLYTICA.......-----MGIKGQEGNCILLLKAFGIS-LFFLSALGIRMGLIVYGMYQDQKFNVRYTDIDYD

: :. : *: *: *. :* * :*****

.........................<-------SIGNAL-PEPTIDE-------->**OOOOOOOOOOOOOOOOOOOOOOOO**

**O1**

........................60........70........80........90........100.......110

| | | | | |

H.SAPIENS VFTDAARFVTEGRSPYLRATYRYTPLLGWLLTPNIYLSELFGKFLFISCDLLTAFLLYRL

R.NORVEGICUS VFTDAARFVTEGRSPYLRATYRYTPLLSWLLTPNVYLSELFGKFLFISCDLLTAFLLYRL

M.MUSCULUS VFTDAARFVTEGRSPYLRATYRYTPLLSWLLTPNVYLSELFGKFLFISCDLLTAFLLYRL

C.ELEGANS VFSDAAKHVSNGGSPFDRATYRYTPALAWILLPVVHFPD-FGKILFCIFDILVAILYFKI

D.MELANOGASTER VVTDGARQVLAGDTPFARHTYRYSPIMAYLQTFNILLHPAWGKLLYATFDLLIATLIYRL

T.BRUCEI IVVDGARHMWNGGSPFDRTTFRYTPLLAALVMPSIWIANPMGKLIFASSDLGAAWYCYGV

E.HISTOLYTICA VYNDASRYLVNGESPYRRATYRYTPLLAEILIPDILLNEQFGKILFSIFDIIIACIQFNL

: *.:: : * :*: * *:**:* :. : : : **::: *: * : :

...................**OOOOOOOOOOOOOOOOOOOOOOOOOOOO**OOOOOOOOOOOOOOOTTTTTTTTTTTTTTTTT

**O1** **T1**

............................120....................................130.......140

| | |

H.SAPIENS LL----------LKGL--------GRRQ--------------ACGYCVFWLLNPLPMAVS

R.NORVEGICUS LL----------LKGL--------GRRQ--------------ACGYCVFWLLNPLPMAVS

M.MUSCULUS LL----------LKGL--------GRRQ--------------ACGYCVFWLLNPLPMAVS

C.ELEGANS MEKDLNETKSETREEM--------KDDQ--------------TMNVVIYWLANPLTAIIS

D.MELANOGASTER VHMEIKSQYQKTVQHLLSKFNRPRDSDQSLDALDERSHPENLARASACFWLYNPLTAVIS

T.BRUCEI LKSF----------AK--------ERSA--------------KWMVSLFILFNPIVLSVS

E.HISTOLYTICA LRQT-----NSFIM----------------------------SLLYTAIWAFNPMSIVIS

: **: :*

TIII IIITT TTTTTTTTTTTTTTTTTO

**I1** **T2**

...........................150.......160................170........180.......190

| | | | |

H.SAPIENS SRGNADSIVASLVLMVLYLIKKR---------LVACAAVFYG-FAVHMKIYPVTYILPIT

R.NORVEGICUS SRGNADSIVASLVLTTLYLIEKR---------LIACAAVFYG-FAVHMKMYPVTYILPIA

M.MUSCULUS SRGNADSIVASLVLSTLYFIEKR---------LIACAAVFYG-FAVHMKMYPVTYILPIA

C.ELEGANS ARGNAESIVAAVVLLNIVLLQKG---------YWKSAALVHGALAIQLKIYPLIYLPSVF

D.MELANOGASTER TRGSGDCFSSFFVILTIYLLLKSEHNVTRSYWLIFGAGLAHG-LVIHLRLYPLLFSLAYY

T.BRUCEI TRGNSDMLVTFMSLMVLSKFARR---------KCYQAAAVLG-FAVHFKIYPIIYALPLT

E.HISTOLYTICA TRGNAEAVVCLFVILTFYFLYKR---------KIWLCSLFFG-LSIHMKIYPVLYSLPLF

:**..: . . : : : : .. * : :::::**: : .

OOOTTTTTTTTTTTTTTTTTIII IIITTTTTTTTTTTTTTTTTTOOOOTTT

**O2** **T3** **I2** **T4** **O3**

.......................................200...............210........220.......230

| | | |

H.SAPIENS LHLLPDRDNDKSLRQFRYTFQACLYELLKRLC-NRAVLLF-VAVAGLTFFALSFGFYYEY

R.NORVEGICUS LHLRPERDSDEGLRLARYSFQARLYDFLKRLC-SWAVLLF-VAIAGLTFLALSFGFYYKY

M.MUSCULUS LHLRPERDDDERLRQARFSFQARLYDFLRRLC-SWAVLLF-VAVAGLTFVALSFGFYYKY

C.ELEGANS LSLSTIGE------------QSCVVNKFKSLVSNWKGFAY-MLVTLTSFAAVVLFFFQIY

D.MELANOGASTER LSLSTRLT------------QTPLDFLCQILRPNKQQLCL-ISGTLISLVAFTWTFYTMY

T.BRUCEI LGVWEQSV-------------AASTNTWRRVVKTAVVVSICALMAAISFAVPTVLCYMKY

E.HISTOLYTICA FCLSNFYP------------SKSFFTKER--------LIA-VFGTAFVLIGLTGYYYYRY

: : : . : : : *

TTTTTTTT TTTTTTIII III-TTTTTTTTTTTTTTTTTOO

**T5** **I3** **T6**

............................240.......250.......260.............270.......280

| | | | |

H.SAPIENS GWEFLEHTYFYHLTRRDIRHNFSPYFYMLYL-TAESKW-----SFSLGIAAFLPQLILLS

R.NORVEGICUS GWEFLEHTYLYHLTRRDIRHNFSPYFYMLYL-TAESKW-----SFTLGIAAFLPQFILLS

M.MUSCULUS GWEFLEHTYFYHLTRRDIRHNFSPYFYMLYL-TAESKW-----SFTLGIAAFLPQFILIS

C.ELEGANS GQLFLDEYLIYHVKRRDLAHNFSPYFYLLYLYEANPTM-----SQIIGLGAFIPQIVLIV

D.MELANOGASTER GWEYIYEAYLYHFVRKDPRHNFSLQFLLQYL-GSASSVAEP--SAILKTLVLAPQFLLIL

T.BRUCEI GQQYLNEAFIYHVYREDHRHNFSPYWLLMYL-NMARRHLGQGVDFSPRLVAFVPQAVVLS

E.HISTOLYTICA GFEFLWETYLYHGTRTDHRHNFSVYWYYLSL-SFDFPK-----STIRSLISFLPQIFCLI

* :: . :** * * **:* : * . : ** . :

OOOOOOOOOOOOO**OOOOOOOOOOOOO**OOOOO OOOOOO OOOOTTTTTTTTTTTTT

**O4** **T7**

..........................290.......300.......310.......320.........330

| | | | |

H.SAPIENS --AVSFAY-YRDLVFCCFLHTSIFVTFNKVCTSQYFLWYLCLLPLVM--P----------

R.NORVEGICUS --AASFAY-YRDLVFCCFLHTSIFVTFNKVCTSQYFLWYLCLLPLVM--P----------

M.MUSCULUS --AASFAY-YRDLVFCCFLHTSIFVTFNKVCTSQYFLWYLCLLPLVM--P----------

C.ELEGANS --FFAFKH-YDDLPFCWFITTFAFVTYNKVCTSQYFVWYIVLLPLLA--H----------

D.MELANOGASTER YLSLSFGQFRQTLPFCIFAVAFVIVTYNSVVTSQYFIWYLAILPLCL--N----------

T.BRUCEI --FVSYKL-RRNTAHACCVQTVLFVAFNKVCTVQYFVWFIPFLAFLFCEPKEVEDDESGG

E.HISTOLYTICA --AIGFRYCKKDIIFCCYLMTCIFVAFNKVYTMQYFIWWFVLLPFVV--P----------

.: .. : :*::*.* * ***:*:: :*.:

TTTTIIIIIITTTTTTTTTTTTTTTTTTOOOOTTTTTTTTTTTTT T

**I4** **T8** **O5** **T9**

...............................340.......350.......360.......370.......380

| | | | |

H.SAPIENS ---LVRM-PWKR---AVVLLMLWFIGQAMWLAPAYVLEFQGKNTFLFIWLAGLFFLLINC

R.NORVEGICUS ---LVRM-PWKR---AVVLLMLWFIGQALWLAPAYVLEFQGKNTFLFIWLAGLFFLLINC

M.MUSCULUS ---LVRM-PWKR---AVVLLLFWFIGQALWLAPAYVLEFQGKNTFLFIWLAGLFFLLINC

C.ELEGANS ---KIMM-SRQL---ALSLMAAWFATQGIWLLAAYLFEFQGWNTFFLMFLASCLFLIANS

D.MELANOGASTER ---NFKMLSWRR---CFGLLFLWLLAQALWLLPAYLLEFHTWNTFYWIGLQGAVFFATNG

T.BRUCEI SGAFKFF-SWVK---ALGVVLMWAATIPLWVTTAVPLEFHGYSDFAKLWIVSCLFFLAMV

E.HISTOLYTICA ---KIIEGALHHIFLTIFIILQYIASYGIWLYYGYELEFKGKNSMFNIFIAGIIVFIANI

. . :: : :*: . :**: . : : : . ..:

---TTTIIIIIITTTTTTTTTTTTTTTTTTTTOOOOOOOOOOOOOOOTTTTTTTTTTTTT

**I5** **T10** **O6** **T11**

......................390.......400........410

| | |

H. SAPIENS SILIQIISHYKEEPL---TER-IKYD

R. NORVEGICUS SILIQIISHYKEDRL---TER-IKYD

M. MUSCULUS SILIQIISHYKEDRL---TER-IKYD

C. ELEGANS FILKQIINHYVP------IVK-PKTD

D. MELANOGASTER YILEQLLSHYGFTQFKISYRK-LL--

T. BRUCEI VLASMLARIAYRVQCTKCSAKSIKVA

E. HISTOLYTICA ILIIWHIYVYSLSDSLRKQKQ-LKLN

: :

TTTTTTTTTTTTIIIIIIIII IIII

**T11** **I6**

Legend of Figure S1.

Amino acid sequence homology between PIG-M from *E. histolytica* and PIG-M from other eukaryotes.

The amino acid sequences were retrieved from the GenBank (NCBI) and Uniprot libraries and compared by BLAST. *E. histolytica* (XM_644988); *H. sapiens (Q9H3S5); R. norvegiens (Q9EQY6); M. musculus (Q99J22); C. elegans (Q17515); D. melanogaster (Q9W2E4); T. Brucei (Q9BPQ5).* Alignment was done using the T-Coffee program [16]. The following potential sites are common to all the sequences: two sites of phosphorylation by phosphokinase C (^74^TYR^76^ and ^141^STR^143^); one site of phosphorylation by casein-kinase 2 (^49^TDID^52^); two sites of N-myristoylation (^5^GQEGNC^10^ and ^28^GIRMGL^33^) that surround the hydrophobic region of the ER sorting signal sequence; one site of O-glycosylation (^251^NLSV^254^). In addition, the potential sites specific to the *E. histolytica* sequence are: two phosphokinase C phosphorylation sites (^269^TIR^271^ and ^402^SLR^404^); one AMPc- and GMPc-dependent protein kinase phosphorylation site (^71^RRAT^74^); three casein kinase 3 phosphorylation sites (^101^SFID^104^, ^244^TRTD^247^ and ^398^SLSD^401^); one tyrosine-kinase phosphorylation site (^47^RYTDIDY^53^); and one N-myristoylation site (^144^GNAEAV^149^).

I: intracytoplamic loop; O: intraluminal loop; T: transmembrane helix.
